# *Pelargonium graveolens* Essential Oil Suppresses Proliferation and Migration and Modulates Mesenchymal-Associated Cellular Functions in Human Endometriotic Cells

**DOI:** 10.3390/cells15080702

**Published:** 2026-04-15

**Authors:** Elif Karakoç, Sezai Berkand Koçak, Kevser Kişifli Köş, Hülya Kayhan, Eda Erdem Şahinkesen, Cemil Can Eylem, Ferda Topal Çelikkan, Emirhan Nemutlu, Pergin Atilla

**Affiliations:** 1 Department of Histology and Embryology, Faculty of Medicine, Hacettepe University, Ankara 06230, Turkey; kocak.berkand@gmail.com (S.B.K.); perginatilla@gmail.com (P.A.); 2 Art De Huile, Teknopol İstanbul, İstanbul 34930, Turkey; mdkevserkos@artdehuile.com (K.K.K.); hulyakayhan@artdehuile.com (H.K.); 3 Faculty of Medicine, Department of Physiopathology, Institute of Health Sciences, Ankara University, Ankara 06230, Turkey; 4 Department of Vaccine Technology, Vaccine Institute, Hacettepe University, Ankara 06230, Turkey; edaerdem97@gmail.com; 5 Department of Analytical Chemistry, Faculty of Pharmacy, Hacettepe University, Ankara 06230, Turkey; cemilcaneylem@gmail.com (C.C.E.); enemutlu@hacettepe.edu.tr (E.N.); 6 Department of Histology and Embryology, Faculty of Medicine, Ankara University, Ankara 06230, Turkey; ferdatopal@gmail.com

**Keywords:** endometriosis, *Pelargonium graveolens*, proliferation, xCELLigence, migration, mesenchymal, apoptosis, organelle, electron microscopy, metabolomics

## Abstract

**Highlights:**

**What are the main findings?**

- PGEO significantly suppresses proliferation and migration while promoting apoptosis in human endometriotic cells.
- PGEO modulates mesenchymal phenotype, cytoskeletal organization, and stress-associated transcriptional responses, accompanied by ultrastructural alterations.

**What are the implications of the main findings?**

- PGEO regulates key cellular functions associated with mesenchymal phenotype associated with endometriosis progression, including proliferation, migration, and cellular stress adaptation.
- These findings support PGEO as a potential modulator of endometriotic cell behavior and a candidate for further investigation in non-hormonal therapeutic strategies targeting endometriosis.

**Abstract:**

Endometriosis is characterized by enhanced cellular proliferation, migration, and resistance to apoptosis, contributing to lesion persistence and progression. Targeting cellular plasticity and mesenchymal-associated functions may therefore represent a promising therapeutic strategy. Here, we investigated the effects of *Pelargonium graveolens* essential oil (PGEO) on proliferation, apoptosis, migration, cytoskeletal organization, transcriptional regulation, and metabolic alterations in human endometriotic 12Z cells. PGEO treatment suppressed proliferative capacity in a concentration-dependent manner and significantly impaired cell migration, accompanied by reduced β-tubulin expression and decreased levels of mesenchymal-associated markers CD73 and CD105. Increased GRP78 expression together with ultrastructural alterations, including cytoplasmic vacuolization and mitochondrial and endoplasmic reticulum changes, indicated activation of cellular stress responses. Although transcriptional analysis revealed increased *CCND1* and *PIK3CA* mRNA levels, these changes did not parallel the observed suppression of proliferation, suggesting compensatory regulatory responses. Untargeted metabolomic profiling revealed alterations in energy metabolism characterized by increased levels of glycolysis-related metabolites, reduced levels of several amino acids including glutamine and histidine, and changes in lipid-associated metabolites. Collectively, these findings demonstrate that PGEO suppresses proliferative and migratory behavior in endometriotic cells while modulating cytoskeletal, transcriptional, and metabolic pathways, highlighting its potential as a candidate for further investigation in endometriosis-targeted therapeutic strategies.

## 1. Introduction

Endometriosis is a chronic estrogen-dependent inflammatory condition characterized by the presence of endometrial-like tissue outside the uterine cavity. It affects nearly 10% of women of reproductive age worldwide, corresponding to nearly 190 million individuals [1]. The exact etiology of endometriosis remains unclear, and several theories have been proposed to explain its pathogenesis. These include retrograde menstruation theory, coelomic metaplasia, immune dysfunction, stem cell migration via blood and lymphatic circulation, and epigenetic alterations [2,3]. The endometriotic cells are known to be resistant to cell death and have the ability to migrate and proliferate in ectopic areas of the body [2,4]. Clinically, endometriosis manifests with a broad range of symptoms including dysmenorrhea, dyspareunia, dyschezia, chronic pelvic pain, and infertility [5]. These symptoms significantly reduce the patients’ quality of life. Current treatment strategies include analgesics, anti-inflammatory drugs, hormonal contraceptives and surgical excision of the endometriotic foci [6,7]. However, these approaches are often associated with recurrence, side effects, and limited long-term efficacy, highlighting the need for alternative therapeutic strategies targeting the underlying biological mechanisms of the disease.

In addition to enhanced proliferative capacity, endometriotic cells exhibit increased migratory and invasive behavior, which contribute to lesion establishment and persistence. These processes are closely associated with cytoskeletal remodeling and epithelial–mesenchymal transition (EMT)-like changes, enabling cells to acquire a more adaptive and motile phenotype. Alterations in cytoskeletal organization and mesenchymal-associated cellular programs are therefore increasingly recognized as key contributors to disease progression.

In this context, natural bioactive compounds, particularly essential oils (EOs), derived from medicinal plants, have attracted attention due to their anti-inflammatory, antioxidant, and immunomodulatory properties. Essential oils are complex mixtures of lipophilic secondary metabolites synthesized by aromatic plants and have been traditionally used for therapeutic purposes [8].

*Pelargonium graveolens*, commonly referred to as rose geranium, is a well-known aromatic plant belonging to the Geraniaceae family [9]. Originating from Africa, it is widely utilized in the cosmetic, pharmaceutical, and food industries due to its fragrance and biological properties. Previous studies have demonstrated that *Pelargonium graveolens* essential oil (PGEO) exhibits anti-inflammatory activity by reducing nitric oxide production and downregulating pro-inflammatory mediators such as COX-2 and iNOS in cellular models. These effects have been attributed to its major constituents, including citronellol, citronellyl formate, linalool, geraniol, isomenthone, and menthone [1,10,11].

Chemically, PGEO is rich in oxygenated monoterpenes, which constitute the majority of its composition, and also contains phenolic acids and flavonoids identified through chromatographic analyses [1,8,12]. In addition, PGEO and its components have been reported to interact with multiple molecular targets, including cyclooxygenase (COX), 5-lipoxygenase (5-LOX), acetylcholinesterase (AChE), angiotensin-converting enzyme 2 (ACE2), and muscarinic acetylcholine receptor 3 (CHRM3), as well as transcription factors such as NF-κB, c-Jun, and Sp1 [13,14,15]. These interactions suggest that PGEO may modulate pathways involved in inflammation, cellular stress responses, and proliferation.

Given its broad biological activity, PGEO represents a promising candidate for modulating cellular processes relevant to endometriosis. However, its effects on endometriotic cell behavior, particularly in relation to proliferation, migration, cytoskeletal organization, and transcriptional regulation, remain insufficiently characterized.

In this study, we hypothesized that PGEO suppresses the proliferative and migratory capacity of endometriotic cells while modulating cytoskeletal organization and mesenchymal-associated transcriptional programs without inducing generalized cytotoxicity.

To define the experimental framework, the half-maximal inhibitory concentration (IC_50_) of PGEO was determined as 6.48 × 10^−7^ M based on real-time impedance analysis. The tested concentration range (1 mM–1 pM) was selected in accordance with previous studies reporting dose-dependent biological activity of *Pelargonium graveolens* essential oil and its major components [16,17].

## 2. Materials and Methods

### 2.1. Chemicals and Reagents

PGEO was obtained from Art De Huile (HLY Aromatherapy, Istanbul, Turkey). The chemical composition of the essential oil was provided by the manufacturer based on GC–MS analysis of the batch used in this study. The major constituents included citronellol (36.38%), geraniol (14.76%), and other monoterpenes such as linalool, citronellyl formate, and isomenthone in lower proportions, consistent with previously reported PGEO composition profiles. Detailed GC–MS composition is provided in Supplementary Table S2 for transparency and reproducibility. The oil was dissolved in DMSO and diluted in culture medium to the desired concentrations prior to treatment. Dimethyl sulfoxide (DMSO, D8418), paraformaldehyde (158127), Triton X-100 (T8787), and anti-beta tubulin antibody (T8328) were purchased from Sigma-Aldrich (St. Louis, MO, USA). MTT reagent (ab211091) was purchased from Abcam (Cambridge, UK).

Anti-CD90 (328102), anti-CD73 (344002), anti-CD105 (800501), DAPI (422801), and Annexin V-FITC/Propidium Iodide (PI) apoptosis detection kit (640914) were from Biolegend (San Diego, CA, USA). Anti-GRP78 antibody (ab212054), and Alexa Fluor 488 secondary antibody (ab150113) were purchased from Abcam (Cambridge, UK). Cell culture medium Prigrow IV (TM004) was purchased from Applied Biological Materials (ABM, Richmond, BC, Canada). Fetal bovine serum (FBS; FBS-16A) was obtained from Capricorn Scientific (Ebsdorfergrund, Germany). L-glutamine (G7513), and penicillin–streptomycin (P4333) were purchased from Sigma-Aldrich (St. Louis, MO, USA).

### 2.2. Cell Culture

The human endometriotic cell line 12Z was purchased from Applied Biological Materials (T0764, ABM, Canada). Cells were cultured in Prigrow IV medium supplemented with 10% fetal bovine serum (FBS), 2% L-glutamine, and 1% penicillin–streptomycin. Cultures were maintained at 37 °C in a humidified incubator with 5% CO_2_. Mycoplasma contamination was assessed using a PCR-based detection kit (Mycoplasma PCR Detection Kit, G238; Applied Biological Materials, ABM, Canada), following the manufacturer’s instructions. All cell cultures were confirmed to be mycoplasma-free before use in experiments (see Supplementary Figure S1).

All experiments were performed using cells at passage 5. For cell treatment, PGEO was dissolved in DMSO and diluted in culture medium to the final concentrations. To ensure consistency, the final DMSO concentration was maintained at 0.1% (*v*/*v*) across all groups. This concentration is widely reported to have negligible effects on cell viability and gene expression [18,19]. In all analyses and figures the “Control” group refers to cells maintained in culture medium supplemented with 0.1% DMSO without PGEO.

### 2.3. MTT Cell Viability Assay

First, 5 × 10^4^ cells were seeded per well into 96-well plates. Once they reached 70–80% confluence, various concentrations of PGEO (1 mM to 1 pM) were applied to the experimental wells (*n* = 8 per dose), while the control wells received only supplemented media. A broad concentration range was initially selected to capture potential concentration-dependent cellular responses across both high and low exposure levels. The cells were treated for 72 h. MTT reagent was added according to the manufacturer’s protocol, and the absorbance was measured at 570 nm with a reference wavelength of 630 nm using a Versamax microplate reader (Molecular Devices, San Jose, CA, USA). Statistical analysis was performed to determine dose-dependent effects on cell viability.

### 2.4. xCELLigence Cell Impedance Analysis

To evaluate the dose- and time-dependent effects of PGEO on the proliferation of 12Z cells, 5 × 10^4^ cells were seeded per well into E-plates, which have integrated gold electrodes for impedance measurement. In brief, doses of PGEO were arranged as 1 mM to 1 pM, with each condition repeated in seven wells (*n* = 7). Control wells were treated with supplemented cell culture media only. Cell impedance was measured every 15 min using the xCELLigence Real-Time Cell Analysis (RTCA) system (version 2.0, Agilent Technologies, Santa Clara, CA, USA). The IC_50_ value of PGEO was calculated from cell index data within the 24–74 h interval, which was selected as the most informative time window for capturing dynamic treatment-related effects. Dose–response curves were generated based on these measurements using the RTCA system.

### 2.5. Flow Cytometric Analysis

Apoptosis induction was assessed using the Annexin V-FITC/Propidium Iodide (PI) detection kit. The 12Z cells were either treated with the IC_50_ concentration of PGEO or supplemented cell culture media for 24 h. After trypsinization, cells were centrifuged, washed with PBS, and stained with Annexin V-FITC and PI in the dark for 15 min. Flow cytometric analysis of CD73, CD90 and CD105 mesenchymal surface marker expression was also performed. Samples were analyzed using a NovoCyte flow cytometer (Agilent Technologies, Santa Clara, CA, USA) and NovoExpress software (version 1.6.3).

### 2.6. Immunofluorescence Analysis

12Z cells were seeded into 8-well chambered slides and cultured until 70–80% confluency. Cells were fixed with 4% paraformaldehyde and permeabilized with 0.2% Triton X-100 for 10 min. After blocking with 5% skimmed milk in PBS to reduce non-specific binding, cells were incubated with primary antibodies (diluted in 5% skimmed milk-PBS at 1:100 dilution) for 3 h. After washing steps with PBS, secondary fluorescent conjugated antibody (at 1:1000 dilution) was applied for 30 min in the dark. Nuclei were counterstained with DAPI for 10 min. Slides were mounted using an anti-fade mounting media and examined using a fluorescence microscope equipped with a digital camera (Leica DMB6B, DFC7000T, LASV3 Wetzlar, Germany). Image acquisition and processing was performed using LASX software (version 3.0.0.15697). At least 3 images per slide were captured, and the corrected total fluorescence intensity (CTCF) was calculated using ImageJ software (version 1.53, National Institutes of Health, Bethesda, MD, USA).

### 2.7. Wound Healing Assay

Briefly, 1 × 10^5^ 12Z cells/per well were seeded into 6-well plates and cultured to 70–80% confluency. A straight linear scratch was made across the monolayer using a sterile 100-µL pipette tip, and detached cells were removed by washing with PBS. The wells were replenished with either supplemented media or the PGEO at its IC_50_ concentration. Wound closure was calculated using a phase-contrast microscope (IX73, Olympus Corporation, Tokyo, Japan) with cellSens Standart software (version 1.18) at predefined 0, 24, and 48 h. All migration assays were performed in biological triplicates. The migration rates were statistically compared between the control and IC_50_-treated groups.

### 2.8. qRT-PCR

Cells were seeded in T75 flasks and divided into control (*n* = 3) and PGEO-treated (IC_50_, *n* = 3) groups. After 24 h of exposure, cells were trypsinized, pelleted, and preserved in TRIzol™ Reagent at −80 °C. Total RNA was isolated using TRIzol™ followed by purification with the FavorPrep™ Blood/Cultured Cells Total RNA Extraction Kit. RNA yield and purity (A260/A280) were assessed using a NanoDrop™ spectrophotometer (Thermo Fisher Scientific, Waltham, MA, USA). Equal amounts of RNA were reverse-transcribed with the Bio-Rad iScript™ cDNA Synthesis Kit. Quantitative real-time PCR was performed using SYBR^®^ Green chemistry (SsoAdvanced™ Universal Supermix, Bio-Rad, Hercules, CA, USA) on a Bio-Rad real-time PCR detection system with gene-specific primers (Table 1). GAPDH was used as the reference gene for normalization. Amplification was performed according to the manufacturer’s recommended cycling conditions. Relative expression was calculated using the 2^−ΔΔCt^ method.

### 2.9. Transmission Electron Microscopy Analysis

For transmission electron microscopy (TEM), the culture medium was removed, and cells were washed with phosphate-buffered saline (PBS). Cells were fixed in 2.5% glutaraldehyde prepared in phosphate buffer for 2 h at room temperature. Following fixation, cells were centrifuged and washed with PBS, and post-fixed in 1% osmium tetroxide for 2 h at room temperature in the dark. Samples were then dehydrated through a graded ethanol series followed by propylene oxide and embedded in epoxy resin. Ultrathin sections were prepared and examined using a transmission electron microscope (HITACHI HT7800, Hitachi High-Technologies, Tokyo, Japan).

### 2.10. Metabolomics

Metabolomic analysis was conducted using gas chromatography–mass spectrometry (GC-MS) to ensure broad coverage of hydrophilic compounds.

Sample Extraction: Following the treatment of 12 Z cells with PGEO and the maintenance of the Control group (*n* = 4 per group), metabolites were extracted from cell pellets using a cold methanol:water (9:1, *v*/*v*) solvent system. Samples were vortexed, shaken at 800 rpm for 20 min, and centrifuged at 15,000 rpm (+4 °C) for 5 min to achieve phase separation. The upper polar phase (methanol/water) containing the hydrophilic metabolites was collected and stored at −80 °C until analysis.

GC-MS Analysis: Polar fractions were vacuum-dried and derivatized via methoximation (methoxyamine hydrochloride) followed by silylation (MSTFA with 1% TMCS). Chromatographic separation was performed on a DB5-MS capillary column (Agilent). Raw data processing, including peak alignment and metabolite identification against the Fiehn Retention Index library, was conducted using MS-DIAL software (version 4.92, RIKEN Center for Sustainable Resource Science, Yokohama, Japan) [20].

### 2.11. Statistical Analysis

Statistical analyses for MTT, xCELLigence, Flow cytometry, immunofluorescence, wound healing, and qRT-PCR analysis were performed using GraphPad Prism version 10 (GraphPad Software, San Diego, CA, USA). Data distribution was evaluated using the Shapiro–Wilk normality test prior to statistical analysis. Parametric data were analyzed using the unpaired Student’s *t*-test for two-group comparisons and one-way ANOVA followed by Tukey’s post hoc test for multiple comparisons. Non-parametric data were analyzed using the Mann–Whitney U test or Kruskal–Wallis test followed by Dunn’s post hoc test, respectively. The results were considered statistically significant at *p* < 0.05.

Metabolomics statistical analyses were performed using the MetaboAnalyst platform (version 6.0). The dataset was normalized using total ion current (TIC) normalization. Unsupervised multivariate analysis was conducted using Principal Component Analysis (PCA) to explore global metabolic variation and to visualize clustering patterns between experimental groups. Metabolites showing a fold change (FC) > 2 and *p*-value < 0.05 were considered significantly altered, and the results were visualized using a volcano plot. *p*-values were adjusted using the Benjamini–Hochberg false discovery rate (FDR) method. VIP scores from PLS-DA and a hierarchical clustering heatmap of the top 50 metabolites ranked by *t*-test significance were generated to identify key metabolites contributing to group discrimination and to visualize metabolic differences between samples.

## 3. Results

### 3.1. PGEO Reduces Proliferative Capacity of 12Z Endometriotic Cells

**Figure 1:**
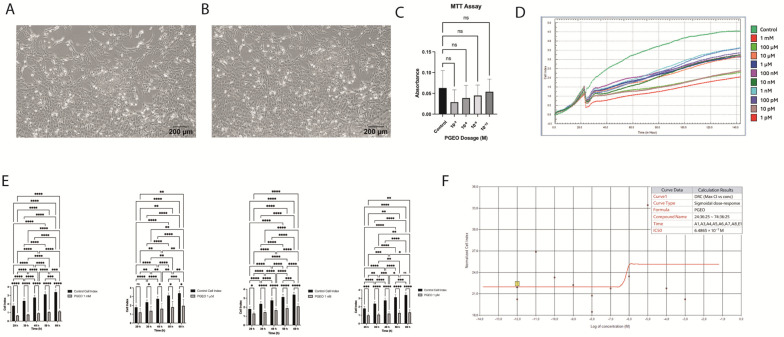
Morphological characterization, viability, and real-time proliferation analysis of 12Z cells treated with *Pelargonium graveolens* essential oil (PGEO). (**A**,**B**) Representative morphology of 12Z cells showing typical small cell size, round nuclei, and prominent nucleoli. Images were acquired at ×100 magnification. (**C**) MTT assay demonstrating the effect of PGEO on cell viability. Absorbance values showed no significant difference between control and treated groups (ns). (**D**,**E**) Real-time proliferation monitoring using the xCELLigence system revealed a suppressive effect of PGEO on 12Z cell growth. Treatment with 1 mM PGEO resulted in a marked and sustained reduction in cell index values at all evaluated time points (29–69 h) compared with control cells. Lower concentrations (1 µM, 1 nM, and 1 pM) produced milder decreases in cell index, indicating a time- and dose-dependent response. (**F**) IC_50_ value calculated from impedance-based proliferation data collected between 24 and 74 h (6.48 × 10^−7^ M). Statistical significance: ns, not significant; * *p* < 0.05; ** *p* < 0.01; *** *p* < 0.001; **** *p* < 0.0001. Scale bars: 50 μm (**B**) and 200 μm (**A**).

Human endometriotic 12Z cells displayed typical spindle-shaped morphology under standard culture conditions and reached 70–80% confluency prior to treatment (Figure 1A,B). In the MTT assay, PGEO-treated cells showed a concentration-dependent reduction in metabolic activity compared with control cells. A reduction in absorbance was most evident at 1 mM, while lower concentrations (1 µM, 1 nM, and 1 pM) showed values comparable to the controls. Statistical significance was not observed (*p* > 0.05) (Figure 1C).

Real-time impedance monitoring using the xCELLigence system revealed a clear inhibitory effect of PGEO on 12Z cell proliferation. Treatment with 1 mM PGEO resulted in a marked and sustained suppression of cell index values across all evaluated time points (29, 39, 49, 59, and 69 h) compared with the controls (*p* < 0.05). Lower concentrations (1 µM, 1 nM, and 1 pM) induced milder reductions in cell index, which became more apparent at later time points, indicating a time- and dose-dependent response (Figure 1D,E). Based on impedance measurements, the IC_50_ value of PGEO was calculated as 6.48 × 10^−7^ M (Figure 1F).

### 3.2. PGEO Promoted Apoptosis in 12Z Cells

**Figure 2:**
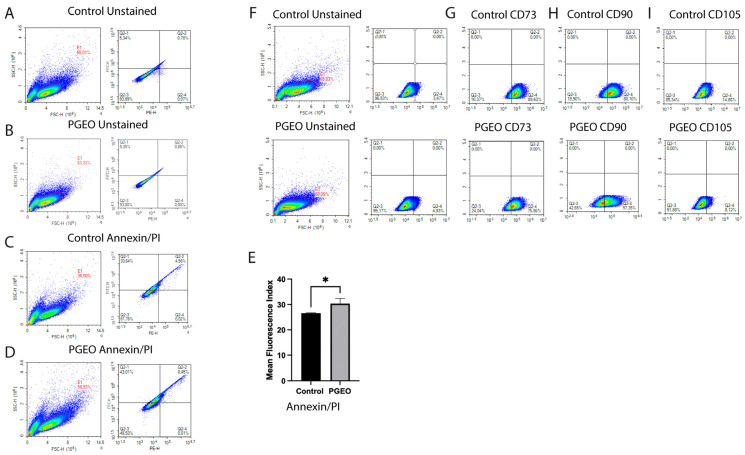
Flow cytometric analysis of apoptosis and mesenchymal-associated surface marker expression together with transcriptional analysis of cell cycle-related genes in 12Z cells following PGEO treatment. (**A**,**B**,**F**) Representative dot plots showing the sequential gating strategy used for flow cytometric analysis. Annexin V/PI staining performed at the IC_50_ concentration of PGEO demonstrated increased apoptotic cell populations and reduced viable cells compared with controls**.** (**C**–**E**) Quantitative analysis confirming a significant increase in total apoptotic cells in PGEO-treated samples (* *p* < 0.05). (**F**–**I**) Flow cytometric evaluation demonstrating reduced surface expression of CD73, CD90, and CD105 in PGEO-treated cells compared with controls.

Apoptotic cell death was evaluated using Annexin V/PI staining following treatment with PGEO at its IC_50_ concentration. Flow cytometric analysis demonstrated a significant increase in the proportion of apoptotic cells in the treated group compared with controls (*p* < 0.05) (Figure 2A–E). Apoptotic cell populations were elevated, accompanied by a reduction in the percentage of viable cells.

### 3.3. PGEO Alters Cytoskeletal Organization and Mesenchymal-Associated Surface Marker Expression

**Figure 3:**
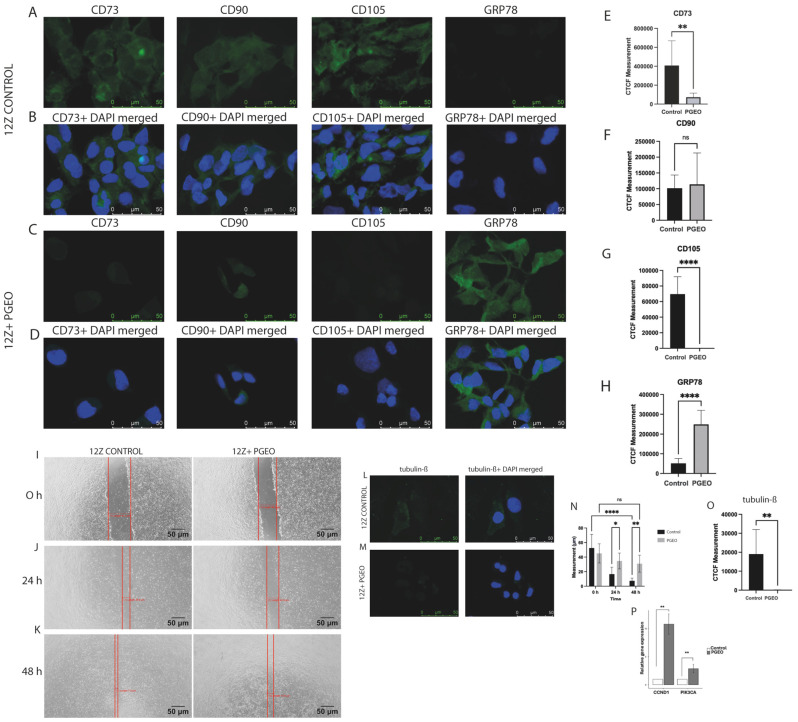
PGEO modulates mesenchymal-associated marker expression, cytoskeletal organization, ER stress response, and migration in 12Z cells. (**A**–**D**) Representative immunofluorescence images of CD73, CD90, CD105, and GRP78 in control and PGEO-treated cells. PGEO reduced CD73 and CD105 immunoreactivity, while CD90 expression remained unchanged. In contrast, GRP78 staining was increased following treatment. (**E**–**H**) Quantification of corrected total cell fluorescence (CTCF) showing significant reduction of CD73 (** *p* < 0.01) and CD105 (**** *p* < 0.0001), no significant change in CD90 (ns), and increased GRP78 expression (**** *p* < 0.0001). (**I**–**K**) Wound-healing assay demonstrating impaired migration in PGEO-treated cells. (**L**,**M**) β-tubulin immunofluorescence showing reduced cytoskeletal organization following PGEO treatment. (**N**) Quantitative analysis demonstrating decreased wound closure in PGEO-treated 12Z cells at 24 and 48 h (* *p* < 0.05 at 24 h; ** *p* < 0.01 and **** *p* < 0.0001 at 48 h, ns: non-specific). (**O**) Quantitative analysis showing reduced β-tubulin fluorescence intensity (** *p* < 0.01). (**P**) Quantitative real-time PCR analysis demonstrating increased *CCND1* and *PIK3CA* mRNA expression in PGEO-treated cells relative to controls. Gene expression levels were normalized to GAPDH and calculated using the 2^−ΔΔCt^ method. Data represent three independent biological replicates (*n* = 3) and are presented as the mean ± SEM (** *p* < 0.01). Nuclei were stained with DAPI (blue). Alexa Fluor 488 (green) was used to detect CD73, CD90, CD105, β-tubulin, and GRP78. Panels (**A**–**D**,**L**–**M**) were acquired at ×1000 magnification, while panels (**I**–**K**) represent phase-contrast images obtained using a ×100 magnification and red vertical lines represent the opposing wound edges for distance measurement. Scale bars: 50 μm.

The expression of mesenchymal-associated surface markers CD73 and CD105 was significantly reduced following PGEO treatment, as evidenced by both immunofluorescence imaging (Figure 3A–D) and CTCF quantification (CD73: *p* < 0.01; CD105: *p* < 0.0001). (Figure 3E–G).

Flow cytometry analysis supported these findings, revealing a decreased surface expression of CD73, CD90, and CD105 in PGEO-treated cells compared with controls (Figure 2F–I). In addition to cytoskeletal alterations and changes in mesenchymal-associated surface marker expression, GRP78 immunofluorescence intensity was significantly increased in PGEO-treated cells (*p* < 0.0001) (Figure 3A–D,H).

Immunofluorescence analysis also demonstrated reduced β-tubulin staining intensity in 12Z cells treated with PGEO at its IC_50_ concentration compared with untreated controls (Figure 3L,M). Quantitative CTCF analysis confirmed a significant decrease in β-tubulin signal intensity (*p* < 0.01) (Figure 3O).

### 3.4. PGEO Impairs Migratory Capacity of 12Z Cells

The wound-healing assay was used to assess the effect of PGEO on 12Z cell migration. At the initial time point (0 h), wound widths were comparable between the control and treated groups (Figure 3I–K). After 24 h, the control cells showed substantial wound closure, whereas PGEO-treated cells exhibited limited migration into the wound area (*p* < 0.05). This difference became more prominent at 48 h, with the treated cells displaying minimal wound closure relative to the controls (*p* < 0.01). Overall analysis of wound closure over the 0–48 h period demonstrated a significant suppression of migratory activity in PGEO-treated cells (*p* < 0.0001) (Figure 3N).

### 3.5. PGEO Modulates Transcription of Cell Cycle Regulatory Genes

Relative gene expression analysis demonstrated that the applied treatment induced statistically significant transcriptional changes in multiple genes compared to the control group. Gene expression levels were calculated using the 2^−ΔΔCt^ method and are presented as the fold change relative to the control group.

*CCND1* gene expression was found to be markedly and statistically significantly increased in the treatment group (*p* < 0.01). Similarly, *PIK3CA* gene expression was significantly elevated following treatment (*p* < 0.01) (Figure 3P).

### 3.6. PGEO Induced Cytoplasmic Ultrastructural Alterations in 12Z Cells

**Figure 4:**
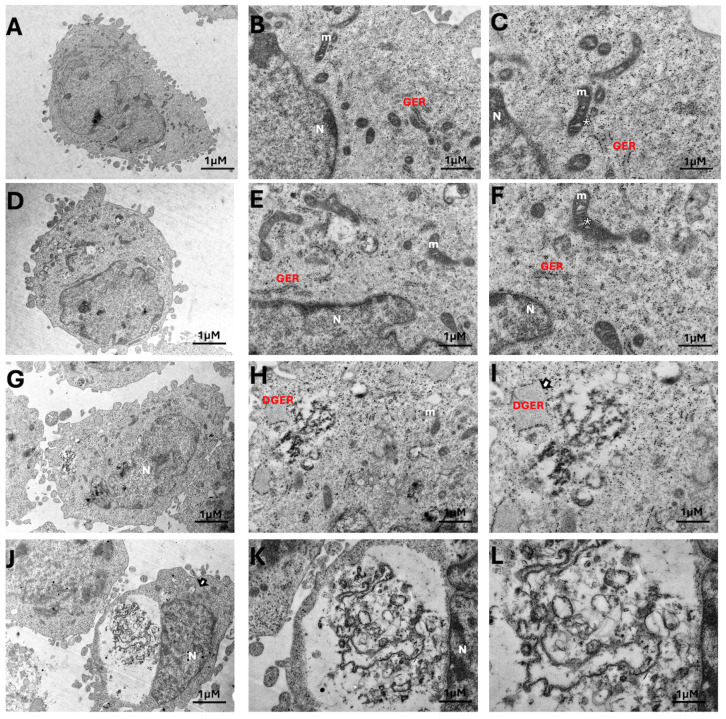
Ultrastructural alterations in 12Z cells following PGEO treatment analyzed by transmission electron microscopy (TEM). (**A**–**F**) Control cells showing preserved ultrastructural organization, characterized by intact nuclear morphology (N), mitochondria with well-defined cristae (m), granular endoplasmic reticulum (GER) and a homogeneously distributed cytoplasmic matrix without prominent vacuolization. (**G**–**L**) PGEO-treated cells demonstrating cytoplasmic alterations including dilatation of granular endoplasmic reticulum cisternae (DGER), accumulation of membranous structures, and formation of large cytoplasmic vacuoles containing heterogeneous membranous material. In several cells, mitochondria appeared displaced toward the cell periphery with partial disruption of mitochondrial cristae. Despite these cytoplasmic alterations, nuclear envelope integrity was preserved and classical double-membrane autophagosomes were not observed. White asterisks indicate mitochondria with preserved cristae in control cells, whereas white arrows indicate dilated endoplasmic reticulum cisternae in PGEO-treated cells. Panels (**A**,**D**,**G**,**J**) represent ×3000 magnification; panels (**B**,**E**,**H**,**K**) represent ×12,000 magnification; and panels (**C**,**F**,**I**,**L**) represent ×20,000 magnification. Scale bars: 1 μm. Ultrathin sections were contrasted with uranyl acetate and lead citrate. Abbreviations: N, nucleus; m, mitochondria; GER, granular endoplasmic reticulum; DGER, dilated granular endoplasmic reticulum.

Transmission electron microscopy (TEM) analysis of 12Z cells treated with PGEO at its IC_50_ concentration demonstrated marked alterations in cytoplasmic ultrastructure compared with untreated controls. Control cells exhibited preserved cellular architecture, characterized by intact nuclear morphology, well-defined mitochondrial cristae, and a homogeneously distributed cytoplasmic matrix without significant vacuolization (Figure 4A–F).

In contrast, 12Z cells treated with PGEO at its IC_50_ concentration exhibited prominent ultrastructural cytoplasmic disruptions. Dilatation of granular endoplasmic reticulum cisternae was occasionally observed. Numerous large cytoplasmic vacuoles were present, some occupying substantial portions of the intracellular space. These vacuoles contained heterogeneous membranous fragments and vesicular structures of variable electron density. Mitochondria appeared redistributed toward the cell periphery in association with expanding vacuolar compartments; mitochondrial cristae disruption was observed.

Despite these cytoplasmic changes, nuclear envelope integrity was maintained, and extensive chromatin condensation, nuclear fragmentation, or karyorrhexis were not evident. Classical double-membrane autophagosomes were not seen. Plasma membrane continuity appeared preserved in the majority of examined cells (Figure 4G–L).

### 3.7. Pelargonium Treatment Promotes Amino Acid Catabolism and Shifts Cellular Energetics

GC-MS-based untargeted metabolomic profiling enabled the annotation of a broad spectrum of intracellular metabolites belonging to multiple biochemical classes. These included amino acids and their derivatives, organic acids associated with central carbon metabolism, nucleotides and nucleobases, carbohydrates and sugar phosphates, fatty acids and lipid-related metabolites, as well as polyamines and redox-related compounds. Together, these metabolite groups reflect key metabolic pathways such as energy metabolism, amino acid metabolism, nucleotide metabolism, and lipid metabolism. The complete list of annotated metabolites is provided in Supplementary Table S1.

**Figure 5:**
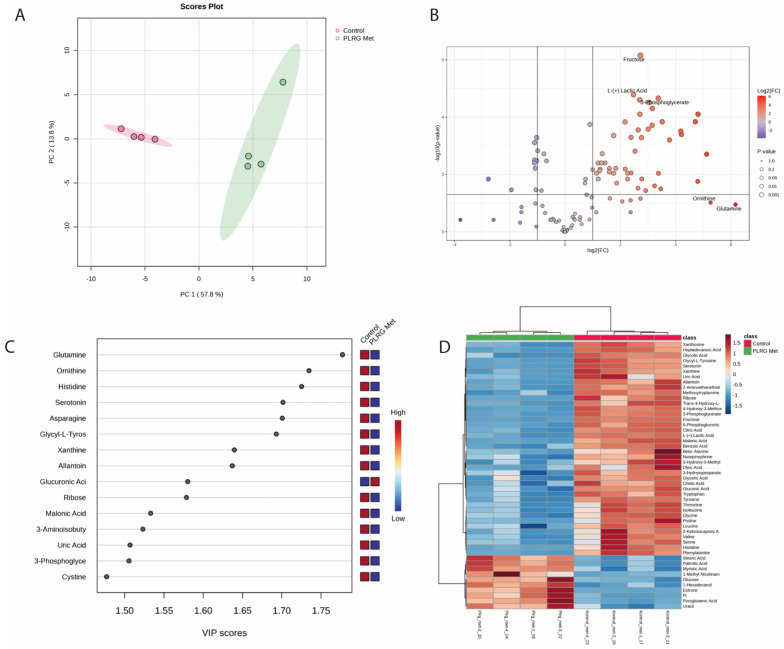
Metabolomic profiling of PGEO-treated 12Z cells. (**A**) PCA score plot showing metabolic separation between control and *Pelargonium graveolens* essential oil (PGEO)-treated cells. (**B**) Volcano plot indicating significantly altered metabolites following PGEO treatment based on fold change and statistical significance (*p* < 0.05). (**C**) VIP score plot highlighting the metabolites contributing most to group discrimination in the PLS-DA model. (**D**) Heatmap with hierarchical clustering illustrating relative abundance patterns of significantly altered metabolites across samples (red: higher, blue: lower levels).

Metabolomic profiling revealed clear treatment-associated metabolic alterations in PGEO-treated 12Z cells. PCA analysis demonstrated a distinct separation between control and PGEO-treated groups, indicating global metabolic remodeling (Figure 5A). Volcano plot analysis identified several metabolites that were significantly altered following treatment (*p* < 0.05) (Figure 5B). PLS-DA-derived VIP scores highlighted key metabolites contributing to group discrimination (Figure 5C). Hierarchical clustering further confirmed distinct metabolic signatures between groups, with coordinated changes in metabolite abundance observed in PGEO-treated cells compared with controls (Figure 5D).

Further analysis revealed reductions in several amino acids, including glutamine, ornithine, asparagine, and histidine. In contrast, increased levels of glycolysis-related metabolites such as 3-phosphoglycerate and lactic acid were detected, together with elevated fructose levels. In addition, long-chain saturated fatty acids were increased in treated cells, along with stress-associated metabolites such as 1-methylnicotinamide. Conversely, purine metabolites including xanthine and uric acid were reduced in PGEO-treated cells.

## 4. Discussion

Endometriosis is a chronic and invasive disorder characterized by increased cell plasticity, enhanced migratory capacity, and altered cellular stress responses. Stromal cells play a central role in lesion formation and persistence through their roles in invasion, angiogenesis, and resistance to apoptotic signals. Although 12Z cells are epithelial in origin, they exhibit mesenchymal-like characteristics commonly associated with stromal cell behavior. From a therapeutic perspective, targeting these mesenchymal-associated cellular behaviors, rather than inducing generalized cellular damage, therefore represents a relevant therapeutic strategy. In this study, PGEO appears to exert such effects by modulating proliferation, migration, cytoskeletal organization, and stress-related signaling in human endometriotic cells.

In line with this concept, PGEO reduced the proliferative activity of 12Z cells as assessed by real-time impedance analysis. The inhibitory effect was concentration-dependent and became more evident over time. In contrast, metabolic activity assessed by MTT did not show significant changes. This discrepancy likely reflects the fundamental differences between the two methods: impedance-based analysis enables real-time monitoring of dynamic cellular behaviors, including changes in adhesion, morphology, and proliferation, whereas MTT primarily reflects endpoint metabolic activity [21,22]. Therefore, xCELLigence analysis may detect early or time-dependent cellular responses that are not captured by endpoint assays such as MTT [23].

In parallel with reduced proliferation, PGEO significantly impaired the migratory capacity of endometriotic cells, as demonstrated by delayed wound closure. This functional alteration was accompanied by decreased β-tubulin expression, suggesting disruption of cytoskeletal organization. Given the essential role of microtubule integrity in cell motility, the reduced β-tubulin signal is consistent with the observed impairment in migratory behavior. However, as no proliferation inhibitor was used in the wound healing assay, the observed delay in wound closure may reflect the combined effects of reduced cell migration and decreased proliferation, rather than migration alone. Supporting these findings, previous studies have shown that geraniol, a major monoterpene component of *Pelargonium graveolens*, inhibits proliferation in colorectal cancer cells [15], while PGEO and its extracts suppress cell growth and induce cell death in multiple cancer cell lines, including MCF-7, Hep3B, HeLa, and breast cancer models [24,25]. Geraniol has also been reported to activate mitochondrial apoptotic pathways in Ishikawa endometrial cancer cells, as evidenced by altered Bax/Bcl-2 balance, increased TUNEL positivity, and upregulation of caspases and cytochrome c [26]. Additional studies have demonstrated the antiproliferative effects of geraniol in lung and skin cancer models [27] and its ability to induce cell cycle arrest and apoptosis in MCF-7 cells [28].

Although 12Z cells are epithelial in origin, they are well-recognized for their invasive, mesenchymal-like characteristics and high migratory capacity, which mimic the behavior of active peritoneal lesions [7,29]. Therefore, we evaluated markers typically associated with cellular plasticity and mesenchymal-like transitions, such as CD73 and CD105, to understand the modulatory effects of PGEO.

In addition to functional changes in proliferation and migration, PGEO selectively affected the expression of mesenchymal-associated surface markers. CD73 and CD105 protein expression levels were significantly reduced, while CD90 expression remained unchanged. CD73 and CD105 have been associated with stromal cell plasticity, migratory behavior, and angiogenic support, whereas CD90 is considered a general stromal marker. This expression pattern suggests that PGEO influences cellular functions associated with mesenchymal-like phenotype. Consistent with this interpretation, geraniol has been shown to suppress key signaling pathways related to proliferation and migration, including K-Ras, MAPK, PI3K, Wnt/β-catenin, and TGF-β, in in vivo endometrial cancer models [30]. Moreover, geraniol inhibits endothelial cell migration and proliferation by suppressing VEGF/VEGFR-2–AKT/ERK signaling and reduces tumor vascularization in vivo [31], further supporting its potential impact on stromal and angiogenic processes.

PGEO also led to increased GRP78 expression, a key regulator of cellular stress responses [32]. Its upregulation may reflect an adaptive stress response rather than overt endoplasmic reticulum dysfunction. In parallel, *CCND1* transcription was increased despite reduced proliferative capacity, suggesting a compensatory attempt to maintain cell cycle progression under stress conditions [33].

In contrast to previous studies reporting PI3K downregulation following geraniol treatment [30], we observed an increase in *PIK3CA* mRNA expression in PGEO-treated 12Z cells. This apparent discrepancy may reflect cell-type-specific or context-dependent transcriptional responses. Importantly, *PIK3CA* expression in this study was evaluated only at the mRNA level, and no protein level or functional pathway analyses were performed. Therefore, the observed upregulation may represent a compensatory transcriptional response under stress conditions rather than direct activation of proliferative signaling pathways. The present study is primarily descriptive in nature, and although changes in ER stress markers, *PIK3CA* gene expression, and metabolic profiles were observed, detailed pathway-level functional analyses were not performed. These findings should be interpreted as preliminary observations requiring further mechanistic investigation.

The ultrastructural alterations observed in PGEO-treated cells parallel the functional changes detected in proliferation and apoptosis assays. Cytoplasmic vacuolization, dilation of granular endoplasmic reticulum cisternae, and disruption of mitochondrial cristae indicate structural perturbations at the organelle level [34]. Nevertheless, preservation of nuclear envelope integrity and the absence of extensive chromatin condensation suggest that, at the 6.48 × 10^−7^ M concentration, PGEO does not induce widespread structural collapse or advanced apoptotic degeneration [35]. These findings point to organelle-level structural alterations associated with reduced cellular functionality, without evidence of plasma membrane rupture characteristic of necrosis [36]. No clear double-membrane autophagosomes were observed under the experimental conditions, although further marker-based analyses would be required for definitive assessment of autophagy. This profile supports a modulatory rather than overtly cytotoxic effect at this concentration, in contrast to studies reporting pronounced cytotoxicity at higher PGEO doses or in cancer models [24,28].

In addition to the phenotypic and transcriptional alterations observed in PGEO-treated cells, metabolomic profiling revealed changes in metabolite levels under the experimental conditions. The metabolomic signature suggested alterations in energy metabolism, amino acid balance, and lipid composition. Specifically, changes in glycolysis-related metabolites, including 3-phosphoglycerate and lactic acid, suggest alterations in glycolysis-related pathways. These findings are in line with previous studies reporting altered glycolytic activity in endometriotic cells [37,38,39]. This metabolic pattern is consistent with the ultrastructural alterations observed in PGEO-treated cells, including mitochondrial cristae disruption and endoplasmic reticulum dilation, which may be associated with organelle-related stress responses. Under such conditions, cells may adjust their metabolic pathways to maintain energy homeostasis [40]. Several amino acids, including histidine, asparagine, glycine, serine, and valine, were significantly decreased following PGEO treatment, indicating changes in amino acid levels under the applied conditions. In addition, changes in lipid-related metabolites, including increased levels of saturated fatty acids such as palmitic acid and stearic acid, suggest alterations in lipid composition that may influence membrane properties and cellular function [37,38].

Overall, these findings indicate that PGEO exerts multi-level effects on endometriotic cells by reducing the proliferative capacity, impairing migratory behavior, altering cytoskeletal organization, and modulating stress-related signaling pathways. Rather than inducing a generalized cytotoxic response, PGEO appears to selectively target cellular features associated with endometriotic cell plasticity and survival, which are central to endometriosis pathophysiology. Although this study is limited to an in vitro model using a single endometriotic cell line, it provides mechanistic insight into the potential of PGEO-derived compounds as modulators of endometriotic cell behavior. Future studies incorporating primary endometriotic cells, in vivo models, and detailed pathway analyses will be necessary to further clarify their therapeutic relevance in endometriosis. The concentrations used in this study may not directly reflect in vivo exposure levels due to pharmacokinetic and bioavailability factors, and therefore the findings should be interpreted within the context of an in vitro model. These findings suggest that PGEO or its bioactive components may have potential as modulators of endometriotic cell behavior. However, further preclinical and clinical studies are required to evaluate their therapeutic applicability and safety.

## 5. Conclusions

PGEO exerts multi-level modulatory effects on endometriotic 12Z cells by impairing proliferation and migration while inducing stress-associated structural and transcriptional alterations. These findings support the further investigation of PGEO as a potential non-hormonal modulator of endometriotic cell behavior.

## Figures and Tables

**Table 1:** Primer sequences used for quantitative real-time PCR (qRT-PCR).

Primer Pair	Sequence (5′ 3′)
CCND1 F	TCTACACCGACAACTCCATCCG
CCND1 R	TCTGGCATTTTGGAGAGGAAGTG
PIK3CA F	CGCCTCTTCTTATCAAGCTCGTG
PIK3CA R	GAAGCTGTCGTAATTCTGCCAGG
GAPDH F	GTCTCCTCTGACTTCAACAGCG
GAPDH R	ACCACCCTGTTGCTGTAGCCAA

## Data Availability

The data presented in this study are available from the corresponding author upon reasonable request.

## Supplementary Materials

The following supporting information can be downloaded at https://www.mdpi.com/article/10.3390/cells15080702/s1, Figure S1: PCR-Based Detection of Mycoplasma in 12Z Cell Line; Table S1: Identified metabolites detected in the untargeted metabolomics analysis of 12Z cells; Table S2: Certificate of analysis.

## Abbreviations

The following abbreviations are used in this manuscript:

PGEO	*Pelargonium graveolens* Essential Oil
CTCF	Corrected Total Fluorescence Intensity
TEM	Transmission Electron Microscopy
IC_50_	Inhibitory Concentration 50%
