# Supplementary material for: Pelargonium graveolens Essential Oil Suppresses Proliferation and Migration and Modulates Mesenchymal-Associated Cellular Functions in Human Endometriotic Cells
Source: Cells. 2026 Apr 15;15(8):702. doi: 10.3390/cells15080702 (PMC13114720; doi:10.3390/cells15080702)
Supplement: Supplementary file 1 [file cells-15-00702-s001.zip › Supplementary Table S1.pdf]

Table S1: Identified metabolites detected in the untargeted metabolomics analysis of 12Z cells.

Metabolite	*Control*	*PLRG Met*	*p value*
1,3-Diaminopropane	1.326 ± 0.598	0.674 ± 0.303	9.97×10^{-2}
1-Hexadecanol	0.635 ± 0.117	1.365 ± 0.131	1.66×10^{-4}
1-Methyl Nicotinamide	0.255 ± 0.082	1.745 ± 0.729	6.65×10^{-3}
2-Aminoethanethiol	1.797 ± 0.240	0.203 ± 0.128	2.32×10^{-5}
2'-Deoxyadenosine	1.093 ± 0.041	0.907 ± 0.275	2.28×10^{-1}
2-Hydroxybutyric Acid	1.737 ± 1.144	0.263 ± 0.139	4.30×10^{-2}
2-Ketoisocaproic Acid	1.408 ± 0.308	0.592 ± 0.140	2.91×10^{-3}
3-Aminoisobutyric Acid	1.833 ± 1.023	0.167 ± 0.049	1.74×10^{-2}
3-Hydroxy-3-Methylglutaric Acid	1.637 ± 0.429	0.363 ± 0.271	2.41×10^{-3}
3-Hydroxypropanoic Acid	1.503 ± 0.183	0.497 ± 0.385	3.24×10^{-3}
3-Phenyllactic Acid	1.745 ± 0.737	0.255 ± 0.100	7.09×10^{-3}
3-Phosphoglycerate	1.824 ± 0.138	0.176 ± 0.063	6.10×10^{-7}
4-Hydroxy-3-Methoxybenzoic Acid	1.733 ± 0.138	0.267 ± 0.044	9.35×10^{-7}
6-Phosphogluconic Acid	1.734 ± 0.179	0.266 ± 0.075	5.32×10^{-6}
Adenosine-5-Monophosphate	0.986 ± 0.835	1.014 ± 0.887	9.65×10^{-1}
Adipic Acid	0.503 ± 0.209	1.497 ± 0.975	9.33×10^{-2}
Alanine	1.615 ± 1.032	0.385 ± 0.131	5.59×10^{-2}
Allantoin	1.896 ± 0.328	0.104 ± 0.105	4.60×10^{-5}
Alpha Ketoglutaric Acid	0.860 ± 0.274	1.140 ± 0.432	3.16×10^{-1}
Alpha-Glucosamine 1-Phosphate	1.205 ± 0.798	0.795 ± 0.737	4.79×10^{-1}
Asparagine	1.931 ± 0.972	0.069 ± 0.062	8.74×10^{-3}
Aspartic Acid	0.655 ± 0.233	1.345 ± 2.457	5.96×10^{-1}
Benzoic Acid	1.301 ± 0.060	0.699 ± 0.080	2.01×10^{-5}
Beta-Alanine	1.655 ± 0.633	0.345 ± 0.138	6.79×10^{-3}
Beta-Glycerolphosphate	1.023 ± 0.276	0.977 ± 0.245	8.08×10^{-1}
Capric Acid	1.264 ± 0.320	0.736 ± 0.194	3.02×10^{-2}
Cholesterol	0.999 ± 0.292	1.001 ± 0.420	9.92×10^{-1}
Cholic Acid	1.392 ± 0.164	0.608 ± 0.217	1.20×10^{-3}
Citric Acid	1.798 ± 0.120	0.202 ± 0.147	2.77×10^{-6}
Citrulline	0.848 ± 0.350	1.152 ± 0.361	2.71×10^{-1}
Cysteinylglycine	1.321 ± 0.437	0.679 ± 0.201	3.72×10^{-2}
Cystine	1.808 ± 0.918	0.192 ± 0.156	1.33×10^{-2}
Cytidine	0.713 ± 0.036	1.287 ± 0.370	2.12×10^{-2}
Cytosine	1.026 ± 0.124	0.974 ± 0.356	7.91×10^{-1}
Galactose	1.676 ± 1.103	0.324 ± 0.062	5.00×10^{-2}
D-Glucose-6-Phosphate	1.186 ± 0.251	0.814 ± 0.502	2.33×10^{-1}
Estrone	0.661 ± 0.007	1.339 ± 0.226	9.69×10^{-4}
Fructose	1.737 ± 0.057	0.263 ± 0.028	6.52×10^{-9}
Fumaric Acid	1.251 ± 0.113	0.749 ± 0.223	7.05×10^{-3}
Gluconic Acid	1.434 ± 0.102	0.566 ± 0.230	4.55×10^{-4}
Glucose	0.634 ± 0.088	1.366 ± 0.242	1.28×10^{-3}

Glucuronic Acid	0.135 ± 0.063	1.865 ± 3.105	$3.08×10^{-1}$
Glutamic Acid	1.723 ± 0.848	0.277 ± 0.210	$1.62×10^{-2}$
Glutamine	1.972 ± 1.802	0.028 ± 0.017	$7.43×10^{-2}$
Glyceraldehyde	0.490 ± 0.281	1.510 ± 2.341	$4.20×10^{-1}$
Glyceric Acid	1.333 ± 0.086	0.667 ± 0.255	$2.58×10^{-3}$
Glycerol	0.983 ± 0.103	1.017 ± 0.075	$6.23×10^{-1}$
Glycerol-1-Phosphate	1.020 ± 0.377	0.980 ± 0.536	$9.07×10^{-1}$
Glycerone Phosphoric Acid	1.766 ± 0.982	0.234 ± 0.105	$2.11×10^{-2}$
Glycine	1.462 ± 0.248	0.538 ± 0.211	$1.29×10^{-3}$
Glycolic Acid	1.724 ± 0.108	0.276 ± 0.247	$3.88×10^{-5}$
Glycyl-L-Tyrosine	1.926 ± 0.282	0.074 ± 0.055	$1.35×10^{-5}$
Heptadecanoic Acid	1.784 ± 0.155	0.216 ± 0.061	$1.44×10^{-6}$
Histidine	1.944 ± 0.556	0.056 ± 0.051	$5.10×10^{-4}$
Hypotaurine	1.570 ± 0.592	0.430 ± 0.182	$1.03×10^{-2}$
Hypoxanthine	0.755 ± 0.270	1.245 ± 0.550	$1.61×10^{-1}$
Inosine	0.502 ± 0.393	1.498 ± 1.124	$1.45×10^{-1}$
Isoleucine	1.427 ± 0.259	0.573 ± 0.153	$1.29×10^{-3}$
L-(+) Lactic Acid	1.695 ± 0.038	0.305 ± 0.107	$3.04×10^{-7}$
Leucine	1.504 ± 0.180	0.496 ± 0.347	$2.11×10^{-3}$
Lysine	1.182 ± 0.632	0.818 ± 0.161	$3.06×10^{-1}$
Malic Acid	1.393 ± 0.150	0.607 ± 0.363	$7.07×10^{-3}$
Malonic Acid	1.839 ± 0.256	0.161 ± 0.047	$1.34×10^{-5}$
Mannose	1.372 ± 0.881	0.628 ± 0.203	$1.51×10^{-1}$
Methionine	1.115 ± 0.272	0.885 ± 0.267	$2.74×10^{-1}$
Methoxytryptamine	1.779 ± 0.129	0.221 ± 0.253	$3.39×10^{-5}$
Methylmalonic Acid	0.724 ± 0.531	1.276 ± 0.042	$8.38×10^{-2}$
Myo-Inositol	1.003 ± 0.148	0.997 ± 0.364	$9.77×10^{-1}$
Myristic Acid	0.741 ± 0.135	1.259 ± 0.069	$4.79×10^{-4}$
N-Acetyl-D-Glucosamine	1.509 ± 0.750	0.491 ± 0.241	$4.16×10^{-2}$
N-Acetyl-D-Mannosamine	0.818 ± 0.440	1.182 ± 0.510	$3.22×10^{-1}$
N-Acetyl-L-Glutamic Acid	0.651 ± 0.271	1.349 ± 0.346	$1.90×10^{-2}$
Norepinephrine	1.544 ± 0.345	0.456 ± 0.334	$3.97×10^{-3}$
Oleic Acid	1.375 ± 0.319	0.625 ± 0.092	$3.99×10^{-3}$
O-Phosphocolamine	1.120 ± 0.157	0.880 ± 0.408	$3.13×10^{-1}$
Ornithine	1.949 ± 1.655	0.051 ± 0.036	$6.17×10^{-2}$
Oxalic Acid	1.071 ± 0.197	0.929 ± 0.604	$6.72×10^{-1}$
Palmitic Acid	0.659 ± 0.102	1.341 ± 0.110	$1.01×10^{-4}$
Palmitoleic Acid	0.645 ± 0.284	1.355 ± 0.570	$6.74×10^{-2}$
Pantothenic Acid	1.245 ± 0.144	0.755 ± 0.224	$1.04×10^{-2}$
Phenylalanine	1.543 ± 0.222	0.457 ± 0.043	$7.30×10^{-5}$
Pi	0.636 ± 0.050	1.364 ± 0.231	$8.37×10^{-4}$
Proline	1.679 ± 0.576	0.321 ± 0.116	$3.62×10^{-3}$
Putrescine	0.413 ± 0.141	1.587 ± 0.728	$1.94×10^{-2}$
Pyroglutamic Acid	0.772 ± 0.030	1.228 ± 0.149	$9.68×10^{-4}$
Pyrophosphate	0.964 ± 0.591	1.036 ± 0.396	$8.46×10^{-1}$

Ribose	1.864 ± 0.396	0.136 ± 0.031	$1.28×10^{-4}$
Sarcosine	1.124 ± 0.229	0.876 ± 0.224	$1.73×10^{-1}$
Serine	1.655 ± 0.544	0.345 ± 0.203	$4.03×10^{-3}$
Serotonin	1.931 ± 0.245	0.069 ± 0.052	$5.88×10^{-6}$
Sorbitol	1.090 ± 0.092	0.910 ± 0.527	$5.28×10^{-1}$
Spermidine	0.285 ± 0.162	1.715 ± 2.670	$3.26×10^{-1}$
Sphingosine	1.676 ± 1.625	0.324 ± 0.212	$1.50×10^{-1}$
Stearic Acid	0.669 ± 0.114	1.331 ± 0.140	$3.31×10^{-4}$
Succinic Acid	0.888 ± 0.124	1.112 ± 0.474	$3.96×10^{-1}$
Threonine	1.562 ± 0.391	0.438 ± 0.189	$2.08×10^{-3}$
Thymine	0.995 ± 0.206	1.005 ± 0.599	$9.75×10^{-1}$
Trans-4-Hydroxy-L-Proline	1.743 ± 0.314	0.257 ± 0.096	$1.01×10^{-4}$
Tryptophan	1.705 ± 0.251	0.295 ± 0.299	$3.58×10^{-4}$
Tyrosine	1.679 ± 0.115	0.321 ± 0.269	$8.97×10^{-5}$
Uracil	0.642 ± 0.184	1.358 ± 0.209	$2.15×10^{-3}$
Urea	0.829 ± 0.599	1.171 ± 0.187	$3.18×10^{-1}$
Uric Acid	1.824 ± 0.712	0.176 ± 0.165	$4.04×10^{-3}$
Uridine 5'-Monophosphate	1.296 ± 1.019	0.704 ± 0.118	$2.92×10^{-1}$
Valine	1.413 ± 0.292	0.587 ± 0.150	$2.38×10^{-3}$
Xanthine	1.897 ± 0.340	0.103 ± 0.132	$6.34×10^{-5}$
Xanthosine	1.636 ± 0.160	0.364 ± 0.123	$1.50×10^{-5}$
