# Supplementary material for: Pelargonium graveolens Essential Oil Suppresses Proliferation and Migration and Modulates Mesenchymal-Associated Cellular Functions in Human Endometriotic Cells
Source: Cells. 2026 Apr 15;15(8):702. doi: 10.3390/cells15080702 (PMC13114720; doi:10.3390/cells15080702)
Supplement: Supplementary file 1 [file cells-15-00702-s001.zip › Supplementary Table S2.pdf]

CERTIFICATE OF ANALYSIS

HES20084 - PELARGONIUM GRAVEOLENS ORG OIL

Pelargonium graveolens L.

Aerial part

Organic Essential Oil

Batch : R200224037

Manufacturing Date : 19 Feb 2024	Best Before Date : 19 Feb 2029	Last Analyse Date : 04 Mar 2024

TEST and METHOD	*VALUE*	*SPECIFICATIONS*
Appearance	*Conforms to standard*	
Colour	*Conforms to standard*	
Direct odour	*Conforms to standard*	
Specific gravity (D20/20)	*0,8910*	*0,8830 / 0,9050*
Refractive Index (ND20)	*1,4670*	*1,4610 / 1,4770*
Optical Rotation at 20°C	*-9,0000*	*-14,0000 / -8,0000 °*
GC profile (area%)	*Conforms to standard*	
GC(area%) Pinene alpha	*0,23*	*%*
GC(area%) Myrcene Beta	*0,17*	*%*
GC(area%) Limonene	*0,15*	*%*
GC(area%) Ocimene Beta Trans	*0,11*	*%*
GC(area%) Rose oxide cis	*1,11*	*%*
GC(area%) Rose oxide Trans	*0,46*	*%*
GC(area%) Menthone	*2,86*	*%*
GC(area%) Isomenthone	*4,75*	*%*
GC(area%) Linalool	*4,69*	*%*
GC(area%) Citronellyl acetate	*0,30*	*%*
GC(area%) Citronellyl formate	*8,06*	*%*
GC(area%) 6,9-Guaiadiene	*0,18*	*%*
GC(area%) Germacrene D	*0,14*	*%*

CERTIFICATE OF ANALYSIS

HES20084 - PELARGONIUM GRAVEOLENS ORG OIL

Pelargonium graveolens L.

Aerial part

Organic Essential Oil

Batch : R200224037

Manufacturing Date : 19 Feb 2024	Best Before Date : 19 Feb 2029	Last Analyse Date : 04 Mar 2024

GC(area%) Geranyl formate	*2,98*	*%*
GC(area%) Terpineol alpha	*0,36*	*%*
GC(area%) Geranyl acetate	*0,45*	*%*
GC(area%) Citronellol	*36,38*	*25,00 / 37,50 %*
GC(area%) Geraniol	*14,76*	*10,00 / 18,00 %*
GC(area%) Geranyl butyrate	*1,09*	*%*
GC(area%) Geranyl tiglate	*0,69*	*%*
GC(area%) 10-epi-gamma-eudesmol	*3,68*	*%*
GC(area%) Phenylethyl tiglate	*0,77*	*%*
