# Supplementary figures and images for: Pelargonium graveolens Essential Oil Suppresses Proliferation and Migration and Modulates Mesenchymal-Associated Cellular Functions in Human Endometriotic Cells
Source: Cells. 2026 Apr 15;15(8):702. doi: 10.3390/cells15080702 (PMC13114720; doi:10.3390/cells15080702)

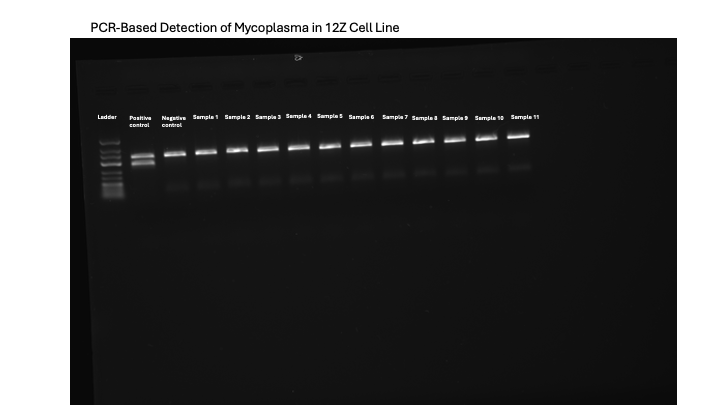

Supplement: Supplementary file 1 [file cells-15-00702-s001.zip › Supplementary Figure S1.tiff]
